# Origin of the Merino Sheep

**Published:** 1881-02

**Authors:** 


### ORIGIN OF THE MERINO SHEEP.

As the ancient Greeks had no cotton or silk, and very little linen, and as sheep's wool was the principal texture from which their clothes were made, they took peculiar care to cultivate such breeds of sheep as produced very fine wool. Such breeds were those of the Greek city of Tarentum, situated on the Tarentine gulf. In order to improve the fine quality of the wool still more, the sheep were covered with clothes in cold weather, as it was found by experience that exposure to cold made the wool coarser. Thus clothing these sheep from generation to generation resulted in a very delicate breed with exceedingly fine wool, according to the law established by Darwin in regard to selection and adaption to exterior conditions.

This product of Greek industry was transmitted by them to the Romans, whose great agricultural author, Colunella, states that his uncle in Spain crossed the fine Tarentine sheep with rams imported from Africa, and obtained a stronger breed, combining the whiteness of the fleece of the father with the fineness of the fleece of the mother, and having obtained such results the race was perpetuated. The absence of other fine textures made these Spanish sheep so valuable that in the beginning of our era they were sold in Rome for $1,000 in gold a head, an enormous price for those times, when money had much more value than now.

When the barbarians invaded Italy these sheep were all exterminated, while the greater portion of the Roman possessions were laid waste. But in the less accessible mountains of Spain the Moors preserved the breed, and it is to them that modern Spain owes the merino sheep, which are the direct descendants of this cross-breed of the Greek and African ancestors referred to. It is a valuable inheritance, too, which that country owes to the combined Greek, Roman and Moorish civilization, and of which our California wool-growers also earn the advantages, by the prosperity of this breed of sheep, which was there a few years ago.

